# Road traffic density and recurrent asthma emergency department visits among Medicaid enrollees in New York State 2005–2015

**DOI:** 10.1186/s12940-022-00885-5

**Published:** 2022-07-28

**Authors:** Tabassum Zarina Insaf, Temilayo Adeyeye, Catherine Adler, Victoria Wagner, Anisa Proj, Susan McCauley, Jacqueline Matson

**Affiliations:** 1 Bureau of Environmental and Occupational Epidemiology, New York State Department of Health, 1203 Corning Tower, Empire State Plaza, Albany, NY USA; 2 Department of Epidemiology and Biostatistics, University at Albany School of Public Health, NY Rensselaer, USA; 3 Department of Environmental Health Sciences, University at Albany School of Public Health, Rensselaer, NY USA; 4 Office of Quality and Patient Safety, New York State Department of Health, Albany, NY USA

**Keywords:** Socio-economic, Traffic, Asthma, Medicaid, Effect modification

## Abstract

**Background:**

Environmental exposures such as traffic may contribute to asthma morbidity including recurrent emergency department (ED) visits. However, these associations are often confounded by socioeconomic status and health care access.

**Objective:**

This study aims to assess the association between traffic density and recurrence of asthma ED visits in the primarily low income Medicaid population in New York State (NYS) between 2005 and 2015.

**Methods:**

The primary outcome of interest was a recurrent asthma ED visit within 1-year of index visit. Traffic densities (weighted for truck traffic) were spatially linked based on home addresses. Bivariate and multivariate logistic regression analyses were conducted to identify factors predicting recurrent asthma ED visits.

**Results:**

In a multivariate model, Medicaid recipients living within 300-m of a high traffic density area were at a statistically significant risk of a recurrent asthma ED visit compared to those in a low traffic density area (OR = 1.31; 95% CI:1.24,1.38). Additionally, we evaluated effect measure modification for risk of recurrent asthma visits associated with traffic exposure by socio-demographic factors. The highest risk was found for those exposed to high traffic and being male (OR = 1.87; 95% CI:1.46,2.39), receiving cash assistance (OR = 2.11; 95% CI:1.65,2.72), receiving supplemental security income (OR = 2.21; 95% CI:1.66,2.96) and being in the 18.44 age group (OR = 1.59;95% CI 1.48,1.70) was associated with the highest risk of recurrent asthma ED visit. Black non-Hispanics (OR = 2.35; 95% CI:1.70,3.24), Hispanics (OR = 2.13; 95% CI:1.49,3.04) and those with race listed as “Other” (OR = 1.89 95% CI:1.13,3.16) in high traffic areas had higher risk of recurrent asthma ED visits as compared to White non-Hispanics in low traffic areas.

**Conclusion:**

We observed significant persistent disparities in asthma morbidity related to traffic exposure and race/ethnicity in a low-income population. Our findings suggest that even within a primarily low-income study population, socioeconomic differences persist. These differences in susceptibility in the extremely low-income group may not be apparent in health studies that use Medicaid enrollment as a proxy for low SES.

**Supplementary Information:**

The online version contains supplementary material available at 10.1186/s12940-022-00885-5.

## Introduction

Asthma exacerbations are considered measures of asthma control and quality of care [1, 2]. When exacerbations result in multiple emergency department (ED) visits, they burden the healthcare system and negatively impact patients’ quality of life [1]. Efforts to reduce such recurrent visits through coordination of services and controlling exposure to indoor and outdoor environmental triggers can decrease health care costs [3–5]. Previous studies have found that residence in urban/inner city areas is associated with increased asthma morbidity such as recurrent ED visits even if it may not be associated with a higher baseline prevalence of asthma [6, 7].

Local road traffic may be an important risk factor for asthma morbidity [8] even if the area meets federal air quality standards for ozone and annual fine particulate matter (PM_2.5_) and does not have exceedances of the 24-hour PM_2.5_ standard as measured by air monitors [9]. Despite declining regional levels of pollution as measured by central monitors, increase in traffic exposures has led to increase in contribution of traffic as a source of air pollution and an increase in the population exposed to such pollution beyond the metropolitan areas [10]. Key components of emissions from traffic sources such as PM_2.5_, volatile organic compounds (VOCs), nitrogen oxides (NO_x_), carbon monoxide (CO), sulfur dioxide (SO_2_), and ammonia (NH_3_), and other ultrafine and nanoparticles, along with other stressors such as noise, may contribute to multiple health outcomes including asthma [11–15]. Measures of proximity to traffic, specifically those accounting for traffic density and truck traffic, may therefore be a cumulative measure of exposure comprising of multiple risk factors of asthma exacerbations than single pollutant measures [9, 16–20].

In the United States, the poor and those belonging to racial/ethnic minorities are more likely to live very close to major highways with increased traffic flow and thus face a high burden of traffic exposure [21]. Trucks and other large vehicles are often routed through these communities and may contribute to a higher burden of traffic [22]. Socio-economic status (SES) characteristics have been found to be associated with increased susceptibility to asthma [23, 24]. Enrollment in Medicaid, a joint federally and state funded program that provides health coverage to over 78.9 million low-income children and adults, is often used as a proxy for SES in the United States [25, 26]. However, few studies have explored the added burden of extremely low-income, race/ethnicity, access to care, and traffic exposures within this primarily low income population.

This study used Medicaid claim and encounter data to determine the risk of asthma exacerbations associated with road traffic density. Additionally, we assessed effect modification of these associations with social determinants of health. We assessed the association between traffic density and the recurrence of ED visits in the New York State (NYS) Medicaid population between 2005 and 2015.

## Methods

### Study Population

**Fig. 1:**
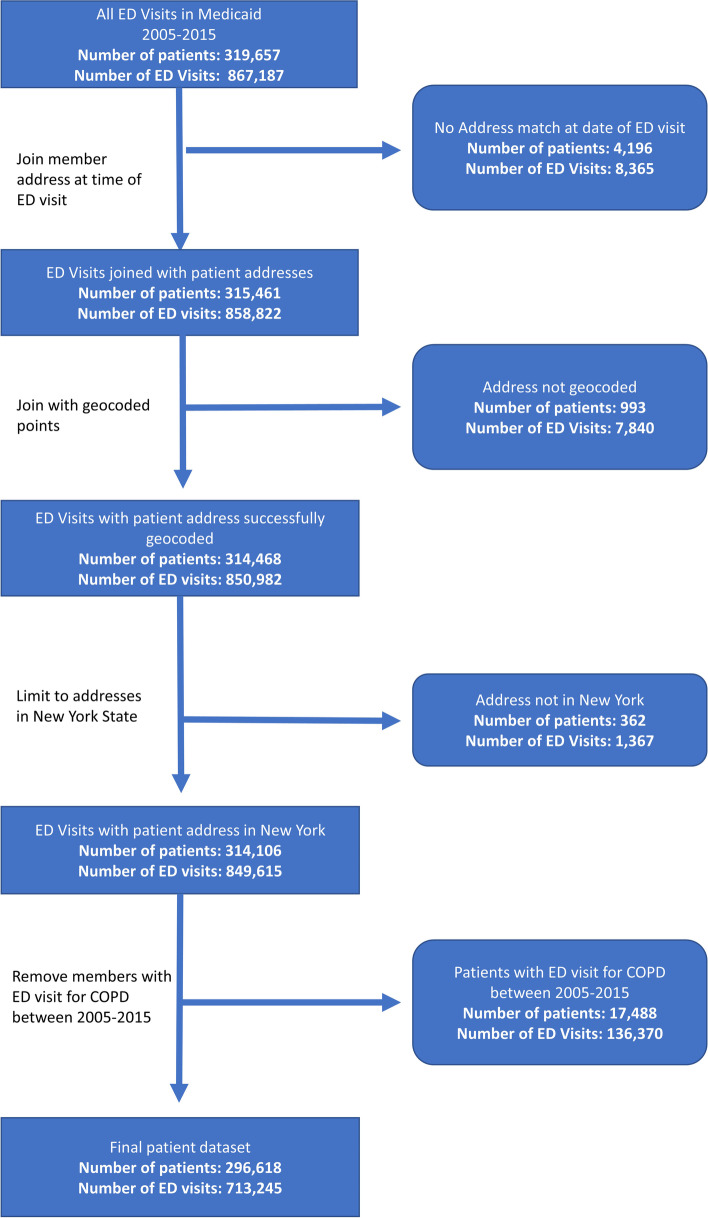
Flow Chart for Exclusion Criteria and Final Analytic Sample for Asthma-related ED visits in New York state Medicaid Population (2005-2015)

The NYS Medicaid program provides comprehensive health coverage to over 7.3 million low-income New Yorkers [27]. Medicaid encounter data, submitted by Medicaid managed care (MMC) health plans, and post-adjudicated fee-for-service (FFS) claim data were used to identify Medicaid enrollees less than 65 years of age who had ED visits with a primary diagnosis of asthma (ICD-9 code 493.xx, ICD-10 code J45; CPT Codes: 99221–99,223, 99,231–99,233, 99,238, 99,239, 99,251–99,255, 99,261–99,263, 99,291) between 2005 and 2015. Medicaid enrollees with a chronic obstructive asthma diagnosis (ICD-9 code 493.2x, ICD-10 code J44.0, J44.1 and J44.9) were excluded (17,488). Most (99.04%) enrollee home addresses at the time of the ED visit were successfully geocoded. Our final study sample consisted of 713,245 ED visits among 296,618 unique Medicaid recipients (Fig. 1).

### Outcome

The primary outcome of interest was a recurrent asthma ED visit within 1 year of the index visit. The number of ED visits within a year was calculated for each patient to create the dichotomous outcome variable. Any patient with two or more asthma ED visits in a year was assigned a value of 1; patients with a single asthma ED visit in a year were assigned a value of 0.

Demographic variables included: age group (≤17, 18–44 and 45–64); sex; race/ethnicity (White non-Hispanic, Black non-Hispanic, Hispanic, and Other); Medicaid coverage program (MMC or FFS); cash assistance; and Supplemental Security Income (SSI) [28]. Medicaid coverage is provided either through a Medicaid managed care health plan (MMC) that manages utilization and quality and is reimbursed by the NYS Medicaid program via monthly capitation payments, or by Medicaid fee-for-service (FFS) which reimburses providers directly for services rendered. NYS Medicaid has increasingly moved to a managed care model and, as such, the proportion of enrollees in FFS (i.e., those in populations excluded or exempt from mandatory MMC enrollment) has decreased over time. NYS Medicaid coverage program was used to assess if the MMC delivery model mitigated enrollees’ risk of recurrent ED visits.

Cash assistance is a measure of state-provided financial assistance and serves as a SES proxy within the Medicaid population. Those receiving cash assistance are the “poorest of the poor”. SSI is a federal financial assistance for the aged, blind, or disabled with limited income and resources. The SSI indicator in our analysis aims to examine the risk of recurrent ED visits among enrollees with disabilities who are more likely to be both medically complex and at elevated risk for multiple ED visits [29]. Note, enrollees may qualify for both cash-assistance and SSI.

### Traffic Exposure

The revised 2016 data of Annual Average Daily Traffic (AADT) from the New York State Department of Transportation were used to create multiple measures of traffic: UWTD (unweighted traffic density), TTD (truck traffic density), and WTD (weighted traffic density). UWTD was an unweighted measure taken directly from the 2016 AADT data and includes the total number of vehicles on a segment of road each day. The TTD is an estimate of truck exposure determined from percentage of trucks for that road segment. Trucks were defined as vehicles belonging to Vehicle Classification Codes F04 (Buses) through F13 (Seven-or-More Axle, Multi-Trailer Trucks) [30]. Finally, the WTD was a weighted estimate calculated by including a multiplier for trucks on a road segment to account for the higher emissions associated with truck traffic. Traffic density was then calculated for each traffic count using the Line Density function within ArcMap (ArcGIS Desktop, Version 10.5, Esri, Redlands, CA) to generate a raster with resolution of 50 m and search radii of 300 m to produce datasets where the value of each raster cell reflected all traffic on the roads within 300 m from the centroid. Thus, the impact of any single roadway on a cell’s traffic density depends on the amount of traffic on that road segment and its distance from the center of the raster cell. The unit for traffic density is the number of vehicles per square meter per day.

Traffic densities and health outcomes data were spatially linked to assign traffic exposure at the individual level based on home addresses. Unique geographic coordinates of the addresses were mapped as points, and traffic density values for the grid within which each point was enclosed were assigned to that point. All addresses outside the specified search radius received a traffic density value of zero (0) and were considered unexposed to vehicle-related emissions at that search distance.

To explore the relationship between different levels of traffic density and recurrent asthma ED visits, tertiles were determined from the statewide distributions of the three traffic density variables and assigned labels of “Low,” “Medium,” and “High” exposure; those individuals with a traffic density value of zero (0) were incorporated into the “Low” exposure referential group. An inverse hyperbolic sine transformation was applied to traffic density counts to evaluate the association between recurrent asthma visits and traffic density as a continuous variable, maintaining no exposure as a legitimate level of exposure to traffic density. The inverse hyperbolic sine transformation can be used to transform heavily right-skewed data containing negative or zero values, approximating a log-transformation for values strictly greater than zero (0) [31]. As the values of traffic density are strictly non-negative, the results of regressions are interpreted as the odds of experiencing recurrent asthma ED visits with a 10 % increase in traffic density.

### Statistical Analysis

The association between recurrent asthma ED visits and traffic density was calculated using median and interquartile range (IQR) since the data were not normally distributed. We calculated the baseline characteristics of the study population to estimate the median and IQR for continuous variables, or percentages of categorical variables in total and by the outcome variable.

Bivariate and multivariate logistic regression analyses were conducted to identify significant factors that affect recurrent asthma ED visits. To evaluate the robustness of the findings, we performed the multivariate regression analysis with each traffic measure separately. The results were represented as the odds ratio (OR) and 95% confidence intervals (CI). Statistical analyses were performed using SAS version 9.4. Confounding was assessed if a 10% change in estimate was observed when a covariate was removed from the model. Effect modification was estimated by adding interaction terms with a traffic measure for selected covariates. All statistical tests were two-sided, and *p* values < 0.05 were considered statistically significant.

## Results

Out of 296,618 Medicaid enrollees with asthma ED visits during the study period, 16.58% had at least one recurrent asthma ED visit within a year. The study cohort was more likely to be female (58.12%), between the ages of 18–44 (42.16%), Black non-Hispanic (29.99%), and be enrolled in MMC (86.07%) (Table 1). The study cohort also lived in areas with a traffic density higher than the state average (Table 2).

**Table 1:** Frequency Distribution of Individuals with M Asthma-related ED visits in New York State Medicaid population (2005–2015)

Variables	Total N (%)
Asthma Emergency Department Visits 1 asthma ED visit ≥2 asthma ED visits	296,618 (100) 247,425 (83.42) 49,193 (16.58)
Sex	
Male Female	124,234 (41.88) 172,384 (58.12)
Age at first encounter	
≤17 18–44 45–64 Missing	118,963 (40.11) 125,066 (42.16) 52,587 (17.73) 2 (0.00)
Race/Ethnicity	
White non-Hispanic Black non-Hispanic Hispanic Other Missing	53,446 (18.02) 88,966 (29.99) 79,351 (26.75) 19,735 (6.65) 55,120 (18.58)
Cash Assistance Yes No	130,731 (44.07) 165,887 (55.93)
SSI	
Yes No	60,353 (20.35) 236,265 (79.65)
Managed Care	
Yes No	255,307 (86.07) 41,311 (13.93)

**Table 2:** Univariate Distribution of Traffic Density Exposure (Number of Vehicles per Square Meter per Day)

	Unweighted Traffic Density		Weighted Traffic Density		Truck Traffic Density	
Measures	Study Population	Statewide Distribution	Study Population	Statewide Distribution	Study Population	Statewide Distribution
Mean	76.00	11.87	89.62	13.91	5.74	1.56
Median	49.00	3.00	57.00	3.00	3.00	1.00
Standard Deviation	84.65	32.73	102.23	38.53	7.23	2.45

Approximately 91% of the ED visits in NYS (combined) occurred among Medicaid recipients living within 300 m of a high WTD area during the study period (Table 3). Since New York City (NYC) is densely populated and therefore its inclusion heavily skews the distribution, we also estimated risks for NYS excluding NYC. When NYC is excluded, about 74% of the ED visits occurred in recipients living within 300 m of a high WTD area, 12% in medium WTD areas, and 15% in low WTD areas. Approximately 54% of the ED visits in NYS occurred among recipients living in a high TTD area. However, when NYC was excluded, only 11% of ED visits occurred among those in a high TTD area.

**Table 3:** Number of Asthma-related ED Visits by Traffic Density in New York State Medicaid Population by T (2005-2015)

Model	Traffic Density @ 300 m		
	Low n (%)	Medium n (%)	High n (%)
Weighted			
NYS	32,661 (4.58)	29,189 (4.09)	651,395 (91.33)
NYS excl. NYC	27,531 (14.52)	22,147 (11.68)	139,960 (73.80)
Unweighted			
NYS	28,579 (4.01)	24,089 (3.38)	660,577 (92.62)
NYS excl. NYC	24,123 (12.72)	18,140 (9.57)	147,375 (77.71)
Trucks only			
NYS	134,503 (18.86)	196,154 (27.50)	382,588 (53.64)
NYS excl. NYC	105,955 (55.87)	63,247 (33.35)	20,436 (10.78)

In the unadjusted analysis, Medicaid recipients who lived within 300 m of a high traffic density area had a statistically significant higher risk for a recurrent asthma ED visit compared to those who lived in a low WTD area for all three models (for WTD model OR = 1.60, 95% CI:1.53,1.67) (Table 4).

**Table 4:** Odds Ratios for the Association between Ranked Traffic Density and Asthma-related ED visits in New York State Medicaid Population (2005-2015)

Model	Unadjusted		Adjusted^a^	
	Medium Traffic Density	High Traffic Density	Medium Traffic Density	High Traffic Density
Unweighted^b^				
NYS	1.05 (0.98, 1.13)	**1.58 (1.51, 1.66)**	1.01 (0.94, 1.09)	**1.29 (1.22, 1.36)**
NYS excl. NYC	0.96 (0.89, 1.04)	**1.26 (1.19, 1.33)**	0.94 (0.87, 1.02)	**1.10 (1.03, 1.17)**
Weighted^c^				
NYS	**1.08 (1.01, 1.15)**	**1.60 (1.53, 1.67)**	1.03 (0.96, 1.11)	**1.31 (1.24, 1.38)**
NYS excl. NYC	1.00 (0.93, 1.07)	**1.27 (1.20, 1.34)**	0.96 (0.89, 1.04)	**1.11 (1.05, 1.18)**
Trucks only^d^				
NYS	**1.35 (1.31, 1.39)**	**1.51 (1.46, 1.55)**	**1.23 (1.19, 1.27)**	**1.32 (1.28, 1.37)**
NYS excl. NYC	**1.17 (1.12, 1.22)**	**1.15 (1.08, 1.22)**	**1.11 (1.06, 1.16)**	1.05 (0.97, 1.12)

^a^Adjusted for age (ref = 18–44), sex (ref = F), cash assistance (ref = No), managed care (ref = No), SSI (ref = Non), race (ref = White non-Hispanic)

^b^Unweighted rankings: low (0–1), medium (2–6), high (7–890)

^c^Weighted for trucks rankings: low (0–2), medium (3–9), high (10–1071)

^d^Trucks only rankings: low (0–1), medium (2–3), high (4–79)

After adjusting for potential confounders (sex, age, race/ethnicity, cash assistance, SSI, and managed care), we observed significantly higher risk for a recurrent asthma ED visit when recipients lived in a high traffic density area in all three models. The risk was higher for medium traffic density areas for truck traffic only in the adjusted models (Table 4). The results were less robust for adjusted models accounting for truck traffic when NYC was excluded.

In both the unadjusted and adjusted models (UWTD and WTD) where Inverse Hyperbolic Sine Transformation was carried out (Table 5), we observed significant risk of recurrent asthma ED visits in both medium and high traffic density areas for NYS (combined) and NYS excluding NYC. In the TTD assessment for NYS (combined), for every 10% change in traffic density, the risk of recurrent asthma ED visits ranged from 7 to 16% and 0–11% for the unadjusted and adjusted models, respectively (Table 5). We ran unadjusted and adjusted models for all three measures of traffic density with similar results in terms of covariates. We therefore only present full results for WTD models (Appendix Table A2). In a multivariate model adjusted for traffic, being male was associated with higher risk of recurrent asthma ED visits. Being of younger or older age had a lower risk of a recurrent asthma ED visit than being in the 18–44 age group. As compared to White non-Hispanics, Black non-Hispanics had the highest risk of recurrent asthma ED visits followed by Hispanics and those of “Other” race/ethnicity categories. Those on cash assistance and recipients of SSI had a higher risk of recurrent asthma ED visits than those who did not receive aid. Those insured by Medicaid FFS were at a higher risk of recurrent asthma ED visits, however the association lost significance after multivariate adjustment.

**Table 5:** Odds ratios for the Association between Traffic Density and Asthma-related ED visits using an Inverse Hyperbolic Sine Transformation model in New York State Medicaid Population (2005-2015)

Model	Log Traffic Density (Unadjusted)	Log Traffic Density (Adjusted)^a^
Unweighted		
NYS	**1.11 (1.11, 1.12)**	**1.07 (1.06, 1.08)**
NYS excl. NYC	**1.07 (1.06, 1.09)**	**1.03 (1.02, 1.05)**
Weighted		
NYS	**1.11 (1.10, 1.12)**	**1.07 (1.06, 1.08)**
NYS excl. NYC	**1.07 (1.06, 1.08)**	**1.03 (1.02, 1.05)**
Trucks only		
NYS	**1.15 (1.14, 1.16)**	**1.09 (1.08, 1.11)**
NYS excl. NYC	**1.10 (1.07, 1.13)**	**1.03 (1.00, 1.07)**

^a^Adjusted for age (ref = 18–44), sex (ref = F), cash assistance (ref = No), managed care (ref = No), SSI (ref = Non), race (ref = White non-Hispanic)

**Fig. 2:**
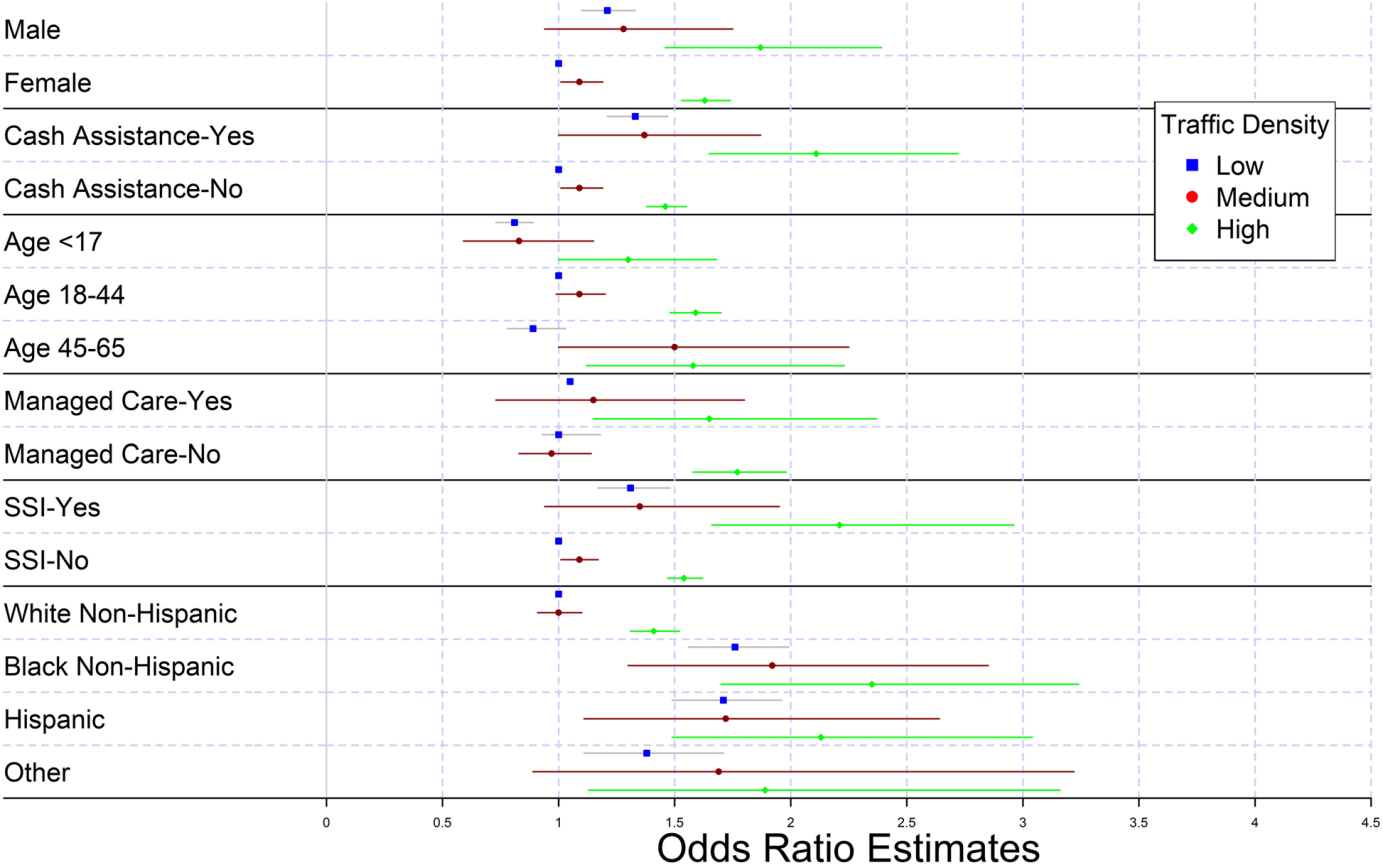
Forest Plot of Odds Ratios and 95% Confidence Intervals for Socio-demographic Factors by Traffic Density in New York State Medicaid Population (2005-2015)

Finally, we assessed effect modification of the risk associated with exposure to traffic density by other covariates (Fig. 2, Table 6). Males were at a higher risk of recurrent asthma ED visits than females at all levels of traffic exposure. Those receiving cash assistance were at a higher risk of recurrent asthma ED visits. Finally, in low traffic areas children less than 17 years old were at the lowest risk of a recurrent asthma ED visit while in high traffic areas adults between 18 and 44 years old had the highest risk of recurrent visits. Those on Supplemental income had a higher risk of recurrent asthma visit. White non-Hispanics were at risk of recurrent asthma visits in only high traffic areas, however those who were Black non-Hispanics, Hispanics or belonged to Other race-ethnicity were at higher risk of recurrent asthma visits at all levels of traffic exposure with increasing risk with higher exposure categories. The risk was highest among Black non-Hispanics.

**Table 6:** Effect Measure Modification of Odds ratios (and 95% Confidence Intervals) for Recurrent Asthma ED Visits with Traffic Exposures by Socio-demographic Factorin NYS Medicaid Population (2005–2015)

	Low Traffic	Medium Traffic	High Traffic
**Sex**			
Male	**1.21 (1.10, 1.33)**	1.28 (0.94, 1.75)	**1.87 (1.46, 2.39)**
Female	Ref.	**1.09 (1.01, 1.19)**	**1.63 (1.53, 1.74)**
**Cash Assistance**			
Yes	**1.33 (1.21, 1.47)**	1.37 (0.997, 1.87)	**2.11 (1.65, 2.72)**
No	Ref.	**1.09 (1.01, 1.19)**	**1.46 (1.38, 1.55)**
**Age**			
< 17	**0.81 (0.73, 0.89)**	0.83 (0.59, 1.15)	**1.30 (1.00, 1.68)**
18–44	Ref.	1.09 (0.99, 1.20)	**1.59 (1.48, 1.70)**
45–64	0.89 (0.78, 1.03)	**1.50 (1.00, 2.25)**	**1.58 (1.12, 2.23)**
**Managed Care**			
Yes	1.05 (0.93, 1.18)	1.15 (0.73, 1.80)	**1.65 (1.15, 2.37)**
No	Ref.	0.97 (0.83, 1.14)	**1.77 (1.58, 1.98)**
**SSI**			
Yes	**1.31 (1.17, 1.48)**	1.35 (0.94, 1.95)	**2.21 (1.66, 2.96)**
No	Ref.	**1.09 (1.01, 1.17)**	**1.54 (1.47, 1.62)**
**Race/ Ethnicity**			
White non-Hispanic	Ref.	1.00 (0.91, 1.10)	**1.41 (1.31, 1.52)**
Black non-Hispanic	**1.76 (1.56, 1.99)**	**1.92 (1.30, 2.85)**	**2.35 (1.70, 3.24)**
Hispanic	**1.71 (1.49, 1.96)**	**1.72 (1.11, 2.64)**	**2.13 (1.49, 3.04)**
Other	**1.38 (1.11, 1.71)**	1.69 (0.89, 3.22)	**1.89 (1.13, 3.16)**

## Discussion

In a large study of almost 300,000 low-income Medicaid enrollees with asthma in New York State from 2005 to 2015, we found that those living in high-density traffic areas were at higher risk of recurrent asthma ED visits compared to those residing in low-density traffic areas. We found that in the WTD model, exposure to high traffic density areas conferred a 31% higher risk of recurrent asthma ED visits than those in a low traffic density area even after adjustment for socio-demographic factors. We also found that racial/ethnic disparities in asthma morbidity persist within this low-income population after adjustment for traffic and other contributing factors. We additionally found evidence of effect modification on the risk due to traffic exposure by certain demographic characteristics. Our study therefore suggests that the interplay of environmental and sociodemographic risk factors may result in a much higher risk of asthma morbidity among specific segments of the most vulnerable. Our study found that exposure to both medium and high traffic density may result in an increased risk of recurrent asthma ED visits. Similar to other studies [32], truck traffic also appears to confer an additional risk as evidenced by the higher risk in the WTD and TTD models in this study. Trucks in the United States are more likely to have diesel engines. As compared with gasoline engines of similar size, diesel engines can generate significantly higher number of particles per distance traveled and therefore may contribute to air pollution at much higher rates [33]. Additionally, a recent study found that there are racial/ethnic disparities in exposure to truck traffic [33]. There may be environmental justice concerns in communities where heavy-duty diesel trucks may transport shipping containers on transport routes through lower socioeconomic status communities [34]. It is likely that the increase in asthma morbidity among high traffic areas may also reflect other environmental conditions in urban inner-city neighborhoods, such as exposure to second-hand smoke, poor building conditions, and proximity to toxic release facilities [7].

TRAP has been suggested to cause physiological damage that can trigger asthma. First, inhaled PM can cause oxidative stress in macrophage and epithelial cells, resulting in airway structural damage. Ultrafine PM < 100 nm in diameter can directly damage mitochondria structurally, impair ATP production, and induce apoptosis in macrophages and epithelial cells. Third, PM has the potential to trigger an immunological cascade response to oxidative stress when antioxidant responses are overwhelmed. These pathways can lead to an adverse respiratory event in individuals with asthma, particularly given evidence for reduced antioxidant enzyme capacity in the lungs and peripheral blood of patients with asthma [35].

Environmental studies have also found that particle concentrations from traffic-related air pollutants (ultrafine PM, black carbon, and CO) generally dissipate by half at around 150 m and decline to background concentrations by 300 m from the upwind source road during the day [36]. This means that individuals who live closest to roads with high traffic density are exposed to the most traffic-related air pollution. However, we did find that medium traffic density areas conferred a high risk as well. We also tested measures between 150 and 300 m and found similar results.

Most studies of traffic-related pollution focus on a specific urban area. In our study, the most urban area was NYC with 70% of its population residing in high traffic density regions. We found the highest effect estimates and highest precision for high truck TD areas in NYC after adjusting for confounders (results not presented). Furthermore, we used traffic density measures across the large and geographically diverse state of New York. We were therefore able to look at risk associated with traffic in the densely populated areas of metropolitan NYC, as well as smaller urban and rural areas across the rest of the state. Our results confirm that TD contributes to significant health risk even in areas with much lower traffic density than large population centers such as NYC.

We also found that those in the 18–44 age group were at a significantly higher risk of recurrent asthma ED visits at baseline levels and were at the highest risk when exposed to high traffic. This may reflect increased risk due to direct occupational exposures to high traffic density compared to those who are younger or older living in similar areas. We found that racial/ethnic disparities in asthma persisted in this low-income population even after adjustment for traffic and other sociodemographic factors. These disparities are likely of a complex origin and cannot be attributed to a single cause. For example, because of the historical systemic discrimination and segregation including redlining, vulnerable racial/ethnic minorities may have lower assets, poorer housing conditions, and poorer health status compared to White non-Hispanics of a similar socio-economic status.

Although males were more likely to have recurrent asthma ED visits in the multivariate adjusted model, we found that females were at a much higher risk of recurrent asthma ED visits when they resided high traffic density areas as compared to females residing in low or medium density areas. A recent study in California also found that the association between the risk of multiple hospital encounters and living near areas of heavy traffic was higher among female children, although that interaction was not statistically significant [37].

In New York State, Medicaid enrollees with very low incomes due to homelessness or disability are eligible for cash assistance. We found that those who received cash assistance were more likely to have asthma exacerbations than those who did not at each level of traffic.

The study results agree with the SES-asthma association suggested by previous studies [23]. Our findings suggest that even within this primarily low-income study population socioeconomic differences persist. These differences in susceptibility in the extremely low-income group may not be apparent in health studies that use Medicaid enrollment as a proxy for low income. Low SES is linked to various health outcomes, with higher rates of morbidity and mortality in chronic diseases such as cardiovascular disease [38], diabetes [39] and chronic obstructive pulmonary disease [40]. In addition, SES may be particularly relevant to asthma in the various pathways through which it may adversely impact asthma health outcomes. Individuals with asthma and lower SES may have higher psychosocial stress and high exposures to both indoor (e.g. mold, cockroaches and tobacco) and outdoor triggers such as pollution [23]. This in turn may increase the risk of asthma exacerbations in such individuals.

Results from this study could be leveraged by Medicaid programs in all 50 states to help identify enrollees with asthma who are most susceptible to the harmful effects of traffic pollution. Understanding the associated risk among minorities and the complex relationship between level of exposure and enrollee characteristics such as sex and receipt of cash assistance, could help Medicaid programs and managed care plans alike facilitate the targeted provision of case management and asthma self-management educational services.

There are some limitations to keep in mind when interpreting the findings of our study. Our measure of traffic-related air pollution was imprecise. We did not have individual measures of exposure, which may lead to misclassification of exposure. We estimated exposure based on distance to nearest roadway and traffic density measures provided by the department of transportation. We also did not have information on the components of traffic related exposures that may lead to the observed associations. Many different components of traffic such as NO_2_, PM_2.5_ or ultrafine particles or noise may contribute to adverse health effects and further research is needed to determine which of these components contribute significantly to the observed associations. We did not have information on other environmental risk factors for asthma morbidity such as indoor housing conditions (e.g., exposure to mold, cockroaches etc.). Datasets on housing conditions or proximity to local sources of non-traffic air pollution in New York only have limited availability and may not be comprehensive. However environmental triggers may also be more prevalent in poorer neighborhoods which would also be likely to have high traffic exposure [41]. Thus, our effect estimates may also reflect the added burden from these environmental exposures in the most vulnerable populations. The Medicaid claims and encounter data used in this study included diagnostic and procedure data. While underreporting is possible, its effects are likely small as Medicaid reimbursement is determined, in part, by enrollee acuity of illness, which is driven by reported diagnoses and procedures. As Medicaid is jointly administered by federal and state governments, financial eligibility criteria vary by state [42, 43] and may affect the generalizability of this study. Additionally, as of 2020 nationwide, there were approximately 7.0 million non-elderly individuals who remained uninsured despite being eligible for Medicaid or the Children’s Health Insurance Program [44]. It remains unclear how low-income individuals enrolling in Medicaid differ from those who do not, though research suggests adults enrolled in Medicaid are more likely to have a regular source of care, report higher rates of preventive care, and use the ED more than uninsured adults [45, 46]. Since we did not have data on individual SES beyond those described in the study, there could be residual confounding due to the imprecise measures used.

As has been reported by others, race/ethnicity reporting in Medicaid data can be unreliable [6, 47]. Eighteen percent of our study subjects had missing race/ethnicity information. This percentage increased toward the end of the study period when more individuals were applying for Medicaid through New York’s online health exchange which collects race and ethnicity as optional fields. However, the proportion of individuals with missing race/ethnicity did not differ from those without missing information in terms of traffic exposures. However, those with missing race/ethnicity data were more likely to be < 17 years of age, and not receiving cash assistance or SSI (Appendix A1). Since those with missing race/ethnicity are likely to be Blacks or Hispanics [48], exclusion of those with missing data would have likely skewed our results towards the null and the underlying associations are probably stronger than reported here.

## Conclusion

Our comprehensive study of asthma morbidity in Medicaid enrollees in New York State found that exposure to high traffic density is associated with increased risk of asthma ED visits. The risk may be even higher in specific subgroups due to physiological susceptibility to higher risk of exposure. Racial and sociodemographic disparities persist in this low-income population even after adjustment for traffic exposures. Our findings suggest that despite significant attention given to asthma in inner city neighborhoods, environmental risk factors such as local traffic can have a higher risk among the most vulnerable. Understanding where this risk is greatest can facilitate the targeted investment of resources to improve patient education and self-management. However, it is only through policies that reduce near road-way pollution exposure including indoor air filtration, relocation of air intakes, and the use of trees and scrubs to serve as vegetative barriers, as well as the re-evaluation of federal emission standards and continued investment in clean diesel programs that the risk will be mitigated. New York State is currently implementing provisions of the Climate Leadership and Community Protection Act, which commits the state to reducing greenhouse gas emissions in a manner that ensures that all New Yorkers, including the most vulnerable, experience the benefits. These findings may be useful in supporting policies which encourage a transition to cleaner transportation options.

## Supplementary Information

**Additional file 1.** . Appendices

## Data Availability

The health data that support the findings of this study are available from the Office of Health Insurance Programs (OHIP), New York State Department of Health (NYSDOH), but restrictions apply to the availability of these data, which were used under license for the current study, and so are not publicly available. Data are however available from the authors upon reasonable request and with permission of OHIP, NYSDOH. The Annual Average Daily Traffic (AADT) data is available from the New York State Department of Transportation Traffic Data Viewer at https://www.dot.ny.gov/tdv

## Abbreviations

AADT: Annual Average Daily Traffic
CI: Confidence Interval
CO: Carbon Monoxide
ED: Emergency Department
FFS: Fee-For-Service
ICD: International Classification of Diseases
IQR: Interquartile Range
MMC: Medicaid Managed Care
NH_3_: Ammonia
NO_X_: Nitrogen Oxides
NYC: New York City
NYS: New York State
OR: Odds Ratio
PM_2.5_: Fine Particulate Matter
SES: Socio-economic Status
SO_2_: Sulfur Dioxide
SSI: Supplemental Security Income
TRAP: Traffic-Related Air Pollution
TTD: Truck Traffic Density
UWTD: Unweighted Traffic Density
VOCs: Volatile Organic Compounds
WTD: Weighted Traffic Density
