# Supplementary material for: Road traffic density and recurrent asthma emergency department visits among Medicaid enrollees in New York State 2005–2015
Source: Environ Health. 2022 Jul 28;21:73. doi: 10.1186/s12940-022-00885-5 (PMC9331590; doi:10.1186/s12940-022-00885-5)
Supplement: Supplementary file 1 — Additional file 1. . Appendices [file 12940_2022_885_MOESM1_ESM.docx]

Table A1: Frequency Distribution of New York State Medicaid Asthma-Related Emergency Department Patients (2005 – 2015) by Race/Ethnicity

| **Variables** | **Race/Ethnicity (Missing)**  **N (%)** | **Race/Ethnicity (Not Missing)**  **N (%)** | **Combined**  **N (%)** | ***P**** |
| --- | --- | --- | --- | --- |
| Traffic Density  Low  Medium  High | 2,420 (4.39)  1,897 (3.44)  50,803 (92.17) | 13,025 (5.39)  10,641 (4.41)  217,832 (90.20) | 15,445 (5.21)  12,538 (4.23)  268,635 (90.57) | <0.0001 |
| NYS  NYC  ROS | 38,702 (70.21)  16,418 (29.79) | 161,892 (67.04)  79,606 (32.96) | 200,594 (67.63)  96,024 (32.37) | <0.0001 |
| Sex  Male  Female | 24,568 (44.57)  30,552 (55.43) | 99,666 (41.27)  141,832 (58.73) | 124,234 (41.88)  172,384 (58.12) | <0.0001 |
| Age at first encounter  ≤17  18 – 44  45 – 64  Missing | 27,044 (49.06)  23,050 (41.82)  5,024 (9.11)  2 (0.00) | 91,919 (38.06)  102,016 (42.24)  47,563 (19.69)  - | 118,963 (40.11)  125,066 (42.16)  52,587 (17.73)  2 (0.00) | <0.0001 |
| Cash Assistance  Yes  No | 16,022 (29.07)  39,098 (70.93) | 114,709 (47.50)  126,789 (52.50) | 130,731 (44.07)  165,887 (55.93) | <0.0001 |
| SSI  Yes  No | 2,230 (4.05)  52,890 (95.95) | 58,123 (24.07)  183,375 (75.93) | 60,353 (20.35)  236,265 (79.65) | <0.0001 |
| Managed Care  Yes  No | 47,112 (85.47)  8,008 (14.53) | 208,195 (86.21)  33,303 (13.79) | 255,307 (86.07)  41,311 (13.93) | <0.0001 |

*Chi-square test comparing missing race/ethnicity versus not missing race/ethnicity

Table A2. Multivariate associations between traffic density, covariates, and recurrent asthma ED visits in NYS Medicaid population 2005-2015

| **Covariates** | **Unadjusted** | **Multivariate Adjusted** |
| --- | --- | --- |
| Traffic (NYS)  High WTD  Medium WTD  Low WTD | **1.60 (1.53, 1.67)**  **1.08 (1.01, 1.15)**  Ref | **1.31 (1.25, 1.38)**  1.03 (0.96, 1.11)  Ref |
| Sex  Male  Female | **1.15 (1.12, 1.18)**  Ref. | **1.24 (1.20, 1.27)**  Ref. |
| Age  <17  18 – 44  45 – 64 | **0.83 (0.80, 0.85)**  Ref.  1.01 (0.97, 1.04) | **0.76 (0.74, 0.78)**  Ref.  **0.85 (0.82, 0.89)** |
| Race/Ethnicity  White non-Hispanic  Black non-Hispanic  Hispanic  Other | Ref.  **1.81 (1.74, 1.89)**  **1.65 (1.58, 1.72)**  **1.45 (1.38, 1.54)** | Ref.  **1.68 (1.61, 1.75)**  **1.52 (1.46, 1.59)**  **1.43 (1.35, 1.51)** |
| Cash Assistance  Yes  No | **1.46 (1.42, 1.49)**  Ref. | **1.28 (1.24, 1.32)**  Ref. |
| SSI  Yes  No | **1.43 (1.39, 1.47)**  Ref. | **1.23 (1.18, 1.27)**  Ref. |
| Managed Care  Yes  No | **0.96 (0.92, 0.99)**  Ref. | 0.97 (0.93, 1.01)  Ref. |

*WTD – weighted traffic density
